# Semipermeable Cellulose Beads Allow Selective and Continuous Release of Small Extracellular Vesicles (sEV) From Encapsulated Cells

**DOI:** 10.3389/fphar.2020.00679

**Published:** 2020-05-21

**Authors:** Gabriela Zavala, María-Paz Ramos, Aliosha I. Figueroa-Valdés, Pablo Cisternas, Ursula Wyneken, Macarena Hernández, Pauline Toa, Brian Salmons, John Dangerfield, Walter H. Gunzburg, Maroun Khoury

**Affiliations:** ^1^ Consorcio REGENERO, Chilean Consortium for Regenerative Medicine, Santiago, Chile; ^2^ Laboratory of Nano-Regenerative Medicine, Faculty of Medicine, Universidad de los Andes, Santiago, Chile; ^3^ Laboratory of Neurosciences, Centro de Investigación Biomédica, Universidad de los Andes, Santiago, Chile; ^4^ Austrianova Singapore Pte Ltd, Singapore, Singapore; ^5^ Department of Pathobiology, Institute of Virology, University of Veterinary Medicine, Vienna, Austria; ^6^ Cells for Cells, Santiago, Chile

**Keywords:** small extracellular vesicles, stem cells, cellulose sulphate microbeads, secretome, Cell-in-a-Box^®^ encapsulation, cell therapy, drug delivery system

## Abstract

The clinical benefit of therapies using Mesenchymal Stem Cells (MSCs) is attributable to their pleiotropic effect over cells and tissues, mainly through their secretome. This paracrine effect is mediated by secreted growth factors and extracellular vesicles (EV) including small EV (sEV). sEV are extra-cellular, membrane encompassed vesicles of 40 to 200 nm diameter that can trigger and signal many cellular responses depending on their cargo protein and nucleic acid repertoire. sEV are purified from cell culture conditioned media using several kits and protocols available that can be tedious and time-consuming, involving sequences of ultracentrifugations and density gradient separations, making their production a major challenge under Good Manufacturing Practices (GMP) conditions. We have developed a method to efficiently enrich cell culture media with high concentrations of sEV by encapsulating cells in semipermeable cellulose beads that allows selectively the release of small particles while offering a 3D culture condition. This method is based on the pore size of the capsules, allowing the release of particles of ≤ 200 nm including sEV. As a proof-of-principle, MSCs were encapsulated and their sEV release rate (sEV-Cap) was monitored throughout the culture and compared to sEV isolated from 2D seeded cells (sEV-2D) by repetitive ultracentrifugation cycles or a commercial kit. The isolated sEV expressed CD63, CD9, and CD81 as confirmed by flow cytometry analysis. Under transmission electron microscopy (TEM), they displayed the similar rounded morphology as sEV-2D. Their corresponding diameter size was validated by nanoparticle tracking analysis (NTA). Interestingly, sEV-Cap retained the expected biological activities of MSCs, including a pro-angiogenic effect over endothelial cells, neuritic outgrowth stimulation in hippocampal neurons and immunosuppression of T cells *in vitro*. Here, we successfully present a novel, cost, and time-saving method to generate sEV from encapsulated MSCs. Future applications include using encapsulated cells as a retrievable delivery device that can interact with the host niche by releasing active agents *in vivo*, including sEV, growth factors, hormones, and small molecules, while avoiding cell clearance, and the negative side-effect of releasing undesired components including apoptotic bodies. Finally, particles produced following the encapsulation protocol display beneficial features for their use as drug-loaded delivery vehicles.

## Introduction

Cell therapy is a constantly growing field as medical needs move toward more targeted and specific solutions. In this context, mesenchymal stem cells (MSCs) represent one of the main actors in basic and translational research. MSCs can be isolated from adult and post-natal tissues, including bone-marrow, adipose tissue, dental tissue, umbilical cord and menstrual fluid (Samsonraj et al., 2017). The therapeutic properties of MSCs mainly reside in their secretions and the paracrine signaling to target cells. The signals themselves are composed of soluble biomolecules (proteins or nucleic acids) or extracellular vesicles (EV) containing them.

EV represents a wide classification of secreted vesicles and comprises microvesicles (MV), apoptotic bodies, microsomes, and sEV among others (Margolis and Sadovsky, 2019). sEV correspond to the smaller in diameter with sizes of > 200 nm, are characterized by the expression of the tetraspanins CD9, CD63, and CD86, and their cellular origin is diverse (Théry et al., 2018). sEV are emerging as key mediators in intercellular communication through horizontal transfer of information *via* their molecular cargo, which includes proteins, DNAs, mRNAs, and miRNAs, that could trigger specific intracellular cascades in the recipient cells (Pegtel and Gould, 2019). For these reasons, the interest nowadays is to obtain large fractions of pure sEV to be used as therapeutic agents without the need for using exogenous cells in patients.

Most commonly, sEV are isolated from cell culture supernatant through methods comprising magnetic particles, immunoaffinity capture-based techniques, ultrafiltration, dialysis, precipitation, size exclusion chromatography (SEC), microfluidics-based isolation techniques, tangential flow filtration (TFF) and ultracentrifugation (Li et al., 2017). Ultracentrifugation is the most commonly used technique, in fact, it is estimated that is used in more than half of isolation protocols for sEV researchers. Differential ultracentrifugation consists in several steps with different centrifugal forces and times that allows the isolation of sEV based on their size and shape and involves the sedimentation of large particles first (such as cells, cell debris, and membrane fragments, apoptotic bodies, and others) that represent a contamination in these cases. After every centrifugation cycle, the supernatant is preserved and the pellet containing the larger vesicles fraction is eliminated. Finally, after the last cycle, sEV are found in the pellet and PBS is usually used for their final resuspension (Gardiner et al., 2016; Li et al., 2017).

Despite the number of different techniques available for sEV isolation, most have significant challenges for upscaling to therapeutic level and for generation of GMP-grade sEV. Therefore, the need for more efficient protocols is justified and could accelerate the translation of sEV into the clinical field. Additionally, the potential use of sEV in patients implies that the challenges should be resolved. For example, how to guide such vesicles to the desired area or how to avoid the rapid clearance that happens in tissues (Liu et al., 2017). For example, some groups have taken a different approach by using hydrogels to directly encapsulate sEV for controlled release in chronic diabetic wounds, which requires long treatments (Shi et al., 2017; Wang et al., 2019) and for cardiac repair that also depends on a continuous supply of the biotherapeutic agent (Han et al., 2019). Some of the limitations of these approaches are the limited number of sEV that can be encapsulated leading to an interrupted supply over longer periods.

Cell encapsulation is a classic technique that has been applied for the delivery of active therapeutic agents from entrapped cells (Acarregui et al., 2013; Gonzalez-Pujana et al., 2017). Their application ranges from insulin release therapy for type 1 diabetes (Orlando et al., 2014) to other life-threatening pathologies, such as cancer (Löhr et al., 2014; Michałowska et al., 2014); also, capsules in general represent the possibility of their localization in a desired area (Dangerfield et al., 2013). The capsule structure must be permeable in order to enable nutrients and waste flux but also the release of the therapeutic agent(s). This makes the development of the encapsulation material as highly challenging. Cellulose sulphate has been developed since more than 20 years (Dautzenberg et al., 1999) and is one of the most used materials due to its inert presentation to the immune system and other relevant properties, such as representing a safe microenvironment for the survival of the cells. Additionally, their handling in the lab does not represent any complications and can be treated as cells and be frozen without damage. Importantly, the use of cellulose sulphate encapsulated human cells is safe in patients as has been demonstrated in two human clinical trials (Löhr et al., 2014) and in a veterinary application (Michałowska et al., 2014).

Encapsulated cells remain viable inside the capsules due to nutrient and waste products exchange with their environment. Moreover, the system presents longer cell viability with the advantageous consequence of a longer secretion time of the molecules of interest (Emerich et al., 2014; Gonzalez-Pujana et al., 2017). This occurs because the cells become contact inhibited once reaching the capsules’ capacity but maintaining its metabolic activity, therefore extending the secretion of the therapeutic molecule. The outflux of many biomolecules, such as insulin, cytokines, antibodies, and enzymes, has been described (Löhr et al., 2014; Salmons and Gunzburg, 2018) but the flux of sEV has not previously been demonstrated.

In this work, we first encapsulate MSCs using semipermeable cellulose beads: Cell-in-a-Box^®^ by Austrianova is a straightforward encapsulation process. The sEV released from encapsulated MSCs derived from the menstrual fluid (MenSCs) (Meng et al., 2007) were characterized. Some of the paracrine properties described for MenSCs include the induction of angiogenic responses *in vitro* and *in vivo*, support the proliferation of CD34^+^ CD133^+^ hematopoietic stem cells *in vitro* (Alcayaga-Miranda et al., 2015a), anti-microbial effect over clinically relevant bacterial strains and protection in an animal model for sepsis (Alcayaga-Miranda et al., 2015b). Safety of MenSCs in patients has been demonstrated in clinical trials (Chen L. et al., 2019).

sEV derived from encapsulated MenSCs (sEV-Cap) were compared with sEV derived from the same cells in a 2D setup and isolated by ultracentrifugation (sEV-2D) in terms of shape, size, and paracrine properties. Here we show that capsule-derived sEV (sEV-Cap) retain these trophic properties *in vitro* meaning that encapsulated cells represent a new and promising technique for the generation and isolation of sEV and their use in the clinical field.

## Materials and Methods

### Ethics Approval

All the procedures were approved by the Ethics Committee of Universidad de los Andes. Samples were obtained with the informed consent of donors.

### MenSCs Isolation

MenSCs were isolated as previously described (Alcayaga-Miranda et al., 2015a). Briefly, menstrual fluid was collected in a menstrual silicone cup (Mialuna^®^, Santiago, Chile) from healthy donors and transferred to a 50-mL conical tube containing 2 mM ethylenediaminetetraacetic acid (EDTA). Mononuclear cells were isolated by a Ficoll-Paque Plus gradient (GE Healthcare, Amersham, UK) and were abundantly washed with PBS 1×. Isolated cells were seeded in T25 flasks and were nourished with high glucose Dulbecco’s modified Eagle’s medium (DMEM) supplemented with 10% fetal bovine serum (FBS), 1% penicillin/streptomycin, 1% amphotericin B, and 1% l-glutamine. Non-adherent cells were discarded the next day. MenSCs were subcultured when reached 80% confluence using 0.05% trypsin-EDTA. Cell cultures were maintained in a humidified incubator at 37°C with 5% CO_2_. All the mentioned cell cultures regents were provided by Thermo Fisher.

### MenSCs Encapsulation (MenSCs-Cap)

Cell-in-a-Box^®^ capsules containing the MenSCs cells were provided by Austrianova Singapore Pte Ltd essentially according to the protocol as previously described (Ortner et al., 2012). Briefly, frozen MenSCs cells from a single donor were sent to Austrianova where they were cultured and trypsinized to give a single-cell suspension. After pelleting, 3.5 × 10^6^ cells were resuspended in Gel8 (proprietary cellulose sulphate solution) and jet-sprayed using an encapsulation machine into a bath of poly-diallyl dimethyl ammonium chloride (pDADMAC). The encapsulated cells were cultured for 1 day in DMEM supplemented with 10% FBS, penicillin/streptomycin, and 2 mM l-glutamine. Each capsule contained approximately 800 to 1,000 cells.

### Capsules Handling

MenSCs-Cap were stored frozen in liquid nitrogen. To defrost, vials were tempered at 37°C in a water bath, and when MenSCs-Cap were settled at the bottom of the vial, the supernatant was eliminated. Next, MenSCs-Cap were transferred to a T25 flask containing culture medium supplemented with additional 50% FBS and incubated for 1 h at 37°C. Next, the medium was eliminated, and MenSCs-Cap were washed with culture medium to finally be maintained with the same culture medium used for MenSCs monolayers. Medium was changed two to three times a week.

### Viability of MenSCs-Cap

Viability was determined after the encapsulation process as a control of the technique and during 16 days to show their behavior over time under cell culture conditions. Post-encapsulation, capsules were frozen and thawed, then, the capsules were incubated in Cell-in-a-Box^®^ Decapsulation Solution as outlined by the supplier (Merck, Cat Nr. CIB002). After the cells had been released from the capsules, cell viability was determined by trypan blue exclusion. The process of encapsulation-freezing, storage, and thawing was used because all the experiments were performed using thawed capsules.

To determine encapsulated cells viability for 16 days, a determined number of MenSCs-Cap was added to a 96-well plate and 10% v/v WST-1 reagent (Quick Cell Proliferation Assay Kit, BioVision, CA, USA) was added to the culture medium. After 2 h incubation at 37°C the supernatant was transferred to a new 96-well plate for absorbance measure at 450 nm/570 nm (Tecan Reader), according to the manufacturer’s guidelines.

### sEV Isolation

For the characterization and comparative sEV studies with MenSCs-Cap, two distinct protocols were used: (1) a commercial Total Exosome Isolation reagent and (2) ultracentrifugation. The Total Exosome Isolation kit (Thermo Fisher) was used according to manufacturer’s instructions. The selection of the isolation protocol was made according to the intended use of sEV: for small volumes, the commercial kit was used and for larger volumes, ultracentrifugation. We denominated sEV-Cap to the EV isolated from encapsulated cells and sEV-2D those EV isolated from MenSCs seeded in monolayers.

MenSCs were cultured in DMEM supplemented with 10% FBS, 1% penicillin/streptomycin, and 1% l-glutamine. When MenSCs monolayers reached 80% of confluence, cells were washed 3 times with PBS 1× and DMEM (phenol red-free) supplemented with 1% penicillin/streptomycin and 1% l-glutamine was added. After 48 h of incubation, the supernatant was recovered and subjected to sequential centrifugation steps for 600*g* for 10 min and 2,000*g* for 10 min, the supernatant recovered correspond to the EV fraction. Next, the EV fraction was centrifuged to 10,000*g* for 30 min to eliminate MV, and finally, the sEV fraction was recovered at 100,000*g* using a TH-641 rotor after 70 min of ultracentrifugation (Thermo Fisher). The supernatant was eliminated, and the resulting pellet was resuspended in PBS 1×, stored at −20°C, and used for experimental procedures.

To obtain supernatant from encapsulated cells, 50 MenSCs-Cap were maintained in 500 µl of DMEM (phenol red-free) supplemented with 1% penicillin/streptomycin and 1% l-glutamine at 37°C and 5% CO_2_, for 48 h. Since this volume is small, we used the commercial kit for sEV isolation. The medium was collected and centrifuged at 600*g* and 2,000*g* for 10 min each to eliminate any cell debris. The supernatant was transferred to a new tube and mixed with Total Exosome Isolation reagent to further incubation at 4°C overnight. Next day, the samples were centrifuged at 10,000*g* for 1 h at 4°C, and the supernatant was eliminated. The pellet, containing the sEV fraction (sEV-Cap), was resuspended in PBS 1× and stored at −20°C for further analysis.

### Quantification of Protein Content of sEV

Protein content from sEV samples were quantified through Bradford assay, measuring absorbance at 590/450 nm using a standard curve of bovine serum albumin (BSA).

### Comparison of sEV Production (sEV-Cap vs sEV-2D)

MenSCs-Cap were added into a 24-well plate in 500 µl (50 capsules per well are equivalent to 50,000 cells approximately). In parallel, 50,000 MenSCs were seeded in another 24-well plate in the same media volume. The serum enriched medium was eliminated for the capsules and cells in monolayer and were washed with PBS 1×, then cells were induced with the same medium described previously. The supernatants were collected after 24, 48, and 72 h and were submitted to sEV isolation using the Total Exosome Isolation kit. The yield was estimated quantifying the protein content through Bradford assay.

### Particle Size and Concentration Characterization With NTA

Isolated EV suspensions were analyzed using the NanoSight NS3000 instrument (Malvern Instruments). The settings were optimized and kept constant between samples for capture settings (laser type, green; camera level, 8; slider shutter, 317; slider gain, 15; temperature, 25°C) and for analysis settings (detection threshold, 3; blur size, auto). Five videos of 60 s each were recorded per sample.

### Flow Cytometry Analyses of sEV

7 × 10^8^ total sEV (quantified by NTA analysis) obtained from MenSCs-Cap or MenSCs monolayers were resuspended in a final volume of 100 µl PBS 1× and 0.5 µl Aldehyde/Sulfate beads (Thermo Fisher, cat. #A37304) were added to the solution and mixed using a benchtop rotator for 10 min. Then, 100 µl of PBS 1× was added to the mixture, and mixing was continued overnight at 4°C. Next day, 100 µl of 1 M glycine in PBS 1× was added, and mixing was continued for 1 h at room temperature. The mixture was spun down at 8,000*g* for 1 min, and the precipitate was resuspended in 100 µl of 10% bovine serum albumin (BSA) in PBS 1× and mixed for 45 min at room temperature. The mixture was spun down at 8,000*g* for 1 min, and the supernatant aspirated. The beads with sEV attached (pellet) were then resuspended to a final volume of 20 µl of 2% BSA in PBS 1× and immunolabeled for CD63, CD81 and CD9 or an isotype control. The sEV bound to beads were incubated with 1 µl of one of the following antibodies: anti-CD63 antibody (BD Pharmingen, cat. 556019), anti-CD81 (BD Pharmingen, cat. 555675), anti-CD9 (BD Pharmingen, cat. 555370) or 10 µl IgG1 isotype control (BD Biosciences, cat. 349040) and mixed for 30 min at room temperature. The mixture was centrifugated at 8,000*g* for 1 min, the supernatant was aspirated, and the pellet was resuspended in 20 µl of 2% BSA in PBS 1×. Then, 1 µl of secondary antibody conjugated with Alexa Fluor 488 (BioLegend, cat. 406626) was added to the samples and isotype control. All samples were mixed at room temperature for 30 min in darkness. The samples were then centrifugated at 8,000*g* for 1 min, the supernatant was aspirated, the pellet was resuspended in 100 µl PBS 1× and washed 2 times with PBS 1×. The expression of sEV markers (CD63, CD81 and CD9) was analyzed using the FACS Canto II flow cytometer (BD Biosciences). Data were analyzed using FlowJo software V10 (Tree Star, Ashland, OR, USA). The flow cytometry data were acquired side by side for both isotype control and samples for each experiment. The gating strategy was similar to the analysis of cells: the beads population was selected from the SSC-A vs FSC-A dot plot and doublets data was eliminated. The data for isotype and the antibodies are shown separately to show the heterogeneity of expression of CD63, CD81, and CD9 in each sample. The MFI (mean fluorescence intensity) values are representative of the entire positive beads.

### Transmission Electron Microscopy (TEM) Analysis

EV visualization of the different fractions by TEM was performed as previously described (Rosenberger et al., 2019). Briefly, EV were stained with uranyl acetate and loaded on a formvar/carbon grid with copper mesh for electron microscopy (Ted Pella, No. 01753-F, US). Images of EV were taken at 60,000× magnification using the Philips Tecnai 12 Biotwin transmission electron microscope with Olympus iTEM software (Laboratorio de Microscopía Electrónica de Barrido SEM, Pontificia Universidad Católica de Chile). Circularity of EV was determined by analyzing these images in ImageJ using the parameters “area”, “perimeter” and “shape descriptors” and the “circularity” measure from the “Analyze Particles” tool. The highest value for circularity is 1.

### Uptake of sEV

sEV-Cap were stained with PKH26 dye (Sigma) to track them in an uptake assay. First, sEV-Cap were mixed with PKH26, previously prepared in Diluent C. PBS 1× was used as control. The samples were incubated for 1 h at room temperature and 1% w/v BSA was added. After incubation, PKH26-stained sEV-Cap were mixed with culture medium and added to previously seeded MenSCs monolayers, as control we used PBS instead of sEV. MenSCs and PKH26-stained sEV were incubated at 37°C for 4 days. Cells were analyzed using an Olympus CX41 microscope and photos were taken for analysis. PKH-26–positive cells were quantified using ImageJ, to show any unspecific stain, we also quantified positive cells in the PBS condition.

### Neurite Growth Assay

The protocol used for neuronal cultures have been described previously and was developed with some modifications (Kaech and Banker, 2006). Briefly, E18 Sprague-Dawley rat fetuses were extracted, and brains were dissected to obtain the hippocampi. Hippocampi were disintegrated with 2.5% trypsin/EDTA and mechanically disaggregated with a glass pipette. 15,000 cells were seeded on poly-L-lysine-coated plates in Minimum Essential Media (MEM) and were incubated in a 5% CO_2_ oven at 37°C for 24 h. The next day, all the media were eliminated and replaced with neurobasal medium, supplemented with 2% B27, 0.03% l-glutamine, and 1% penicillin/streptomycin antibiotic. Same day, 3 µg of total protein of sEV were added to the medium and left for 5 days.

### Immunostaining and Neurite Growth Analysis

Neurons maintained for 5 days *in vitro* (DIV) were fixed and dehydrated in a 4% paraformaldehyde (PFA)–4% sucrose solution for 15 min at room temperature. Cells were permeabilized for 5 min at room temperature in 0.25% Triton X-100 in PBS, washed twice with PBS, and incubated for 30 min with PBS containing 10% BSA for blocking. Cells were incubated overnight at room temperature with the primary antibody anti-MAP2 (Abcam). After washing 3 times with PBS, cells were incubated with the secondary Alexa Fluor 555-conjugated anti-mouse antibody (Life Technologies A21429) diluted 1/5,000 in PBS containing 3% BSA for 45 min at room temperature in darkness. Cells were washed twice with PBS and mounted with ProLong Gold Antifade Reagents containing 4′,6-diamidino-2-phenylindole dihydrochloride (DAPI). Photographs were taken in a Nikon epifluorescence microscope and analyzed in the ImageJ program (NIH). For neurite length and number analysis, the Sholl analysis was used (https://imagej.net/Sholl_Analysis).

### *In Vitro* Tube Formation Assay

Angiogenic potential of sEV-Cap was evaluated through an *in vitro* tube formation assay as described (González et al., 2015). Human umbilical vein endothelial cells (HUVEC) were seeded in 24-well plates (6 × 10^4^ cells per well) previously coated with 250 µl Matrigel^®^ growth factor reduced (GFR) (BD Biosciences). EGM-2 medium was used as positive control and DMEM (without FBS) as negative control. 1 µg of sEV were suspended in DMEM and added to HUVEC. Cells were incubated at 37°C for 5 h, and tube formation was examined with a phase-contrast microscope. Five representative images were captured per well using an Olympus U-RFL-T camera. Quantification of tube formation was analyzed using WimTube software (Wimasis GmbH, Munich, Germany) and the parameters evaluated were total tube length, total loops, and covered area.

### Immunosuppression Assay

The capacity of sEV-Cap to suppress T cells proliferation was evaluated as previously described (González et al., 2015). First, human peripheral blood mononuclear cells (PBMC) were isolated from healthy donors by Ficoll density-gradient centrifugation at 400*g* for 30 min. PBMC were stained with 1 µM carboxyfluorescein succinimidyl ester (CFSE, Thermo Fisher) and treated with 1 µg sEV-Cap. PBMC were maintained in Roswell Park Memorial Institute (RPMI) medium supplemented with 10% FBS, 1% l-glutamine, 1% nonessential amino acids (NEEA), 100 mM sodium pyruvate, 25 mM β-mercaptoethanol, and 15 mg/ml phytohemagglutinin (PHA) for lymphocytes activation, when indicated. After 72 h, PBMC were recovered and stained with anti-CD45 and anti-CD3 antibodies (BD Pharmingen, cat. 55548 and 555333, respectively) for analysis in FACS Canto II Flow cytometer (BD Biosciences) using FlowJo software V10 (Tree Star, Ashland, OR, USA). The percentage of immunosuppression was determined as described previously (Killer et al., 2017).

### Statistics

All assays were performed at least in duplicate or triplicate as indicated. Values are shown as mean ± SD, and statistical significance was estimated using Student’s unpaired *t* test or ANOVA test. P < 0.05 was considered as statistically significant. The software GraphPad Prism 5.0b was used for statistical analysis.

## Results

### Efficient Encapsulation of Viable MenSCs

**Figure 1:**
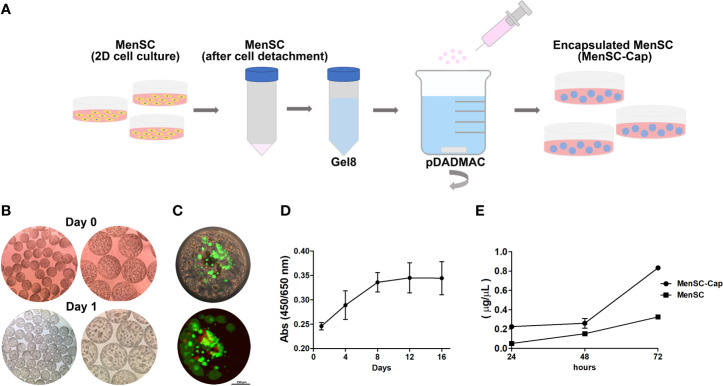
Characterization of MenSCs-Cap. **(A)** Graphic representation of the encapsulation process. MenSCs are cultured in standard conditions, after detaching, cells are mixed with the Gel8 solution. Next, the cellular suspension is jet-sprayed into a bath of poly-diallyl dimethyl ammonium chloride (pDADMAC). The encapsulated cells are maintained in cell culture medium or can be frozen. **(B)** Cellulose capsules containing MenSCs at the same day of encapsulation and after 24 h. **(C)** Live Dead staining shows cell distribution inside the capsule after the defrosting protocol. Live (green) and death (red) cells are shown. **(D)** Cell proliferation of MenSCs-Cap while maintained in standard cell culture conditions measured through absorbance at 450 nm of WST-1 reduction (n = 2). **(E)** sEV production from MenSCs seeded in a standard 2D well plate compared with MenSCs-Cap (n = 2).

Austrianova’s Cell-in-a-Box^®^ encapsulation process is a straightforward process. After MenSCs expansion in a 2D condition, cells were sub-cultured and mixed with Gel8 (proprietary cellulose sulphate solution) and posteriorly, the cell suspension was added dropwise into a bath of pDADMAC. Machine generated capsule sizes are in the range of 750 µm ± 25 µm and can be easily manipulated, frozen, cultured, and maintained in standard cell culture conditions (**Figure 1A**). Capsules were observed under a traditional optic microscope and cells were visualized as denser areas inside the capsule (**Figure 1B**). Encapsulation is a safe process, but some levels of cell apoptosis or necrosis may occur with some cell types as Live Dead staining showed (**Figure 1C**), in fact, viability was close to 65% after the encapsulation protocol but cells remained viable over time as measured by a WST-1 assay (**Figure 1D**), which measures the reduction of tetrazolium salt into formazan by mitochondrial enzymes. Additionally, we compared the efficiency of sEV production of MenSCs and MenSCs-Cap and determined a higher production from encapsulated cells during a period of 24 to 72 h (**Figure 1E**).

### Isolation of sEV From Encapsulated MenSCs (sEV-Cap)

One of the strategies for the production of sEV is the continuous release of vesicles from a carrier, in this case, correspond to encapsulated MenSCs. This could lead to a decrease in the processing time of large volumes of supernatants, but before proposing this alternative, we must describe the characteristics of the sEV generated by encapsulated cells. For that, the supernatant was submitted to the mentioned isolation protocols in order to concentrate the EV and proceed with the characterization.

**Figure 2:**
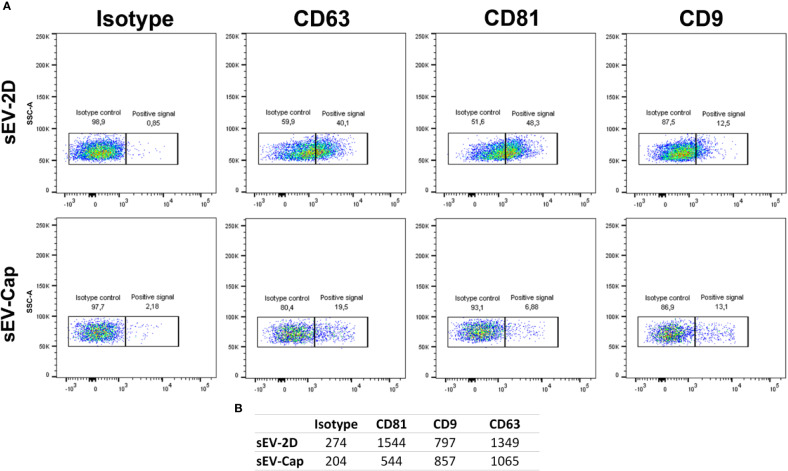
Expression of classic sEV markers in sEV-cap and sEV-2D. **(A)** Flow cytometry characterization of sEV-Cap and sEV isolated from 2D-cultured MenSCs shows that are positive for CD63, CD9, and CD81, characteristic markers for sEV. **(B)** Mean fluorescence index from the isotype controls and the markers analyzed.

As a first step, we evaluated the expression of described sEV surface markers: CD63, CD9 and CD81 (Théry et al., 2018). The analysis showed that sEV isolated from 2D or encapsulated cells were positive for CD63, CD81 and CD9, but differences were detected for CD81 with higher MFI in sEV-2D with respect to sEV-Cap (**Figures 2A, B**, **Supplementary Figure 4**). Considering that the same number of particles were used for the analysis (according to the NTA determination) we can infer that sEV isolated from encapsulated cells expressed lower levels of CD81 than sEVs isolated from cells in 2D. This could be due to a different vesicle population secreted by these cells or these differences can rely on the number of vesicles present in each fraction. Even though the same number of particles were used in the experiment, we cannot discard the presence of contamination in the sEV-Cap due to the isolation protocol (commercial kit), that could underestimate the expression of the different proteins evaluated.

**Figure 3:**
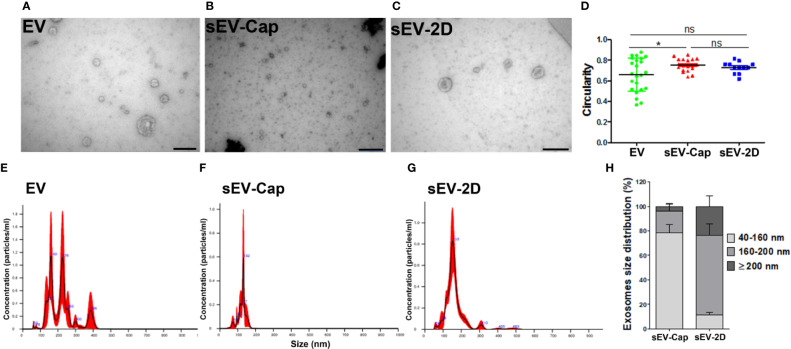
Characterization of sEV-Cap. Shape and size characterization of EV. Representative images from TEM analysis of **(A)** EV, **(B)** sEV-Cap, and **(C)** sEV-2D. Scale bar = 200 nm. **(D)** Circularity of EV respect to their origin. *P  <  0.05 Student’s *t* test, comparison was made between two respective conditions. NTA analysis shows the size distribution of **(E)** EV (8.83 × 10^7^ particles/mL), **(F)** sEV-Cap (1.36 × 10^7^ particles/mL), and **(G)** sEV-2D (4.76 × 10^7^ particles/mL). **(H)** Size distribution of sEV isolated from capsules by ultracentrifugation and isolation from 2D cell culture by the same technique (percentage of particles), data are shown as mean ± SD. There are statically significant differences between the percentage’s distribution: 40–160 nm and 160–200 fractions, for the ≥ 200 nm, there were no differences (*P < 0.05 unpaired Student’s *t* test). ns, not significant.

In order to compare the purity and quality of the samples obtained by different methods, vesicles from several fractions were analyzed through TEM and Nanoparticle Tracking Analysis (NTA). TEM analysis showed the characteristic cup-shape of EV, sEV-Cap, and sEV-2D (**Figures 3A–C**). Additionally, we determined the circularity of the vesicles in order to analyze whether the isolation technique altered the shape of the sEV. As expected, there was a variety of shapes in EV due to their heterogeneous composition and origins. sEV-Cap and sEV-2D presented similar circularity confirming the validity of the isolation techniques (**Figure 3D**). Next, the different fractions were analyzed by NTA (**Supplementary Figures 1****–****3**), EV diameters were close to 218.7 nm ± 75.4 nm, presenting a varied distribution in the size of the vesicles. sEV-2D enriched fraction had sizes around 162.1 ± 54.2 nm, but with the presence of larger vesicles which could represent EVs contamination (**Figures 3E–G**). On the other hand, sEV-Cap size was 123.9 ± 21.8 nm, with a narrower distribution compared to sEV-2D, showing the purity of the sEV released from the porous capsules. Finally, with the NTA data we determined the percentages of sEV according to their size, observing interesting differences in the distribution of vesicles between 40 and 200 nm. For the 40 to 80 nm, 80 to 120 nm, and 120 to 160 nm fractions, there was a higher percentage in sEV-Cap, but in the 160- to 200-nm range sEV-2D contains the higher fraction. Interestingly the fraction of 40 to 160 nm was significantly higher in sEV-Cap, with 78.5% ± 16.5% versus the 11.11% ± 5.51% for sEV-2D. Moreover, in both sEV-Cap and sEV-2D, there was a contamination of vesicles with sizes ≥ 200 nm but in sEV-Cap was significantly lower confirming that encapsulation favors the liberation of sEV with low or absent MV contamination (**Figure 3H**).

### Functional Properties of sEV-Cap *In Vitro*

The optimization and development of more efficient techniques for sEV isolation aims to facilitate the access of these cellular products for their applications in research and translation to the clinic. A novel protocol not only needs to show the capacity for optimizing the process but also the quality of the sEV obtained. Hence, it is crucial to demonstrate the functionality of the isolated vesicles by the new protocol and to compare it with the ones obtained by standard methods. Therefore, we evaluated whether sEV-Cap are internalized by cells in order to produce their *in vivo* effect. MenSCs monolayers were incubated with PKH26-stained sEV, and it was determined that cells were capable of internalizing the sEV-Cap in a 59.7% ± 6% directly from the supernatant of capsules. These results confirmed that endocytosis signals in the vesicles surface were functional (**Supplementary Figure 5**).

### sEV-Cap Induce Pro-Angiogenic Responses in a Tubule Formation Assay

**Figure 4:**
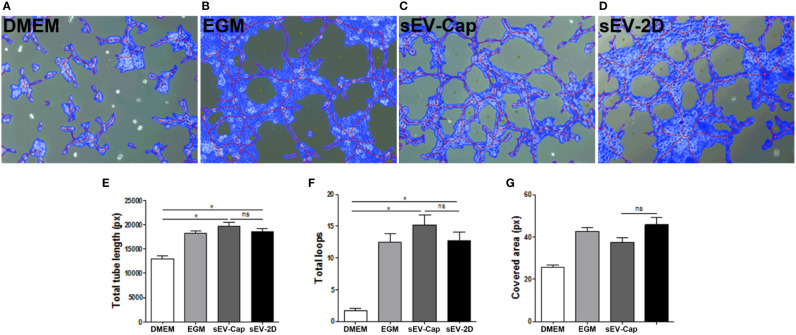
sEV-Cap induces an angiogenic response. sEV elicit a pro-angiogenic response in HUVEC in a tubule formation assay. **(A)** DMEM, serum deprived, as negative control. **(B)** EGM, positive control. **(C)** sEV-Cap. **(D)** sEV-2D. Quantification of **(E)** total tube length, **(F)** total loops, and **(G)** covered area shows that sEV-Cap induce a response similar to sEV-2D (n = 2), and three microscope fields per condition were analyzed for each assay. *P < 0.05 one-way ANOVA. ns, not significant.

As MenSCs and other MSCs are recognized as trophic mediators *in vitro* and in their native niche, we sought to evaluate whether the purified sEV contained these properties as well by performing different functional assays. First, we evaluated the potential of sEV-Cap and sEV-2D to induce a pro-angiogenic response (**Figures 4A–D**), performing a tubule formation assay evaluated by the quantification of total tube length, total loops, and covered area. The effect of both sEV-Cap and sEV-2D were comparable among them in all 3 parameters analyzed (**Figures 4E–G**) and respect to EGM-2, the positive control (**Figure 4B**). These results indicated that sEV retain the trophic abilities of parental MenSCs.

### sEV-Cap Promotes Neuritic Outgrowth in Hippocampal Neurons

**Figure 5:**
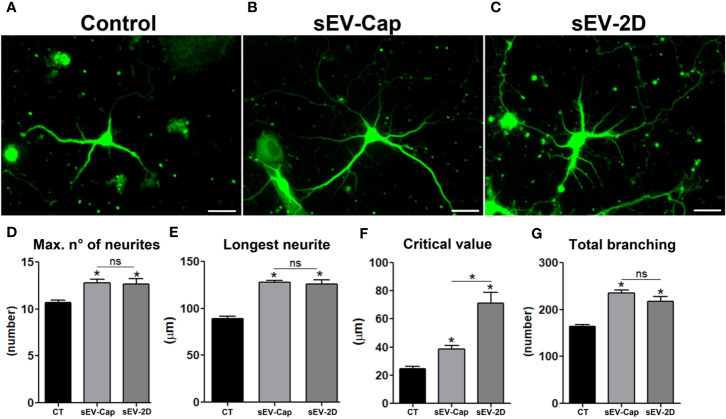
sEV-Cap promotes neurite outgrowth. Rat hippocampal cortical neurons were treated with sEV and neurite elongation was evaluated. **(A)** Control, **(B)** sEV-Cap **(C)** sEV-2D, scale bar = 10 µm. The effect of sEV was evaluated through Sholl analysis; **(D)** Maximum number of neurites, **(E)** longest neurite, **(F)** critical value, and **(G)** total branching (n = 3), 20 neurons were analyzed per condition. *P < 0.05 one-way ANOVA. ns, not significant

Another tested scenario was the potential of MenSCs-derived sEV to induce neuritic growth. To confirm this property, primary cultures of rat hippocampal neurons were treated with either sEV-Cap or 2D-sEV during neurites elongation phase (**Figure 5**). We determined that the presence of the sEV induced a significant increase in the number of neurites (**Figure 5C**) with no differences between sEV-Cap and 2D-sEV. The same trend was observed for the longest neurite and total branching (**Figures 5E–G**), showing that sEV from MenSCs contained growth factors that transduced a cellular signal into the cytoskeleton, promoting the elongation of neurites. Remarkably, the critical value was lower for sEV-Cap respect to 2D-sEV, meaning that the ramifications were closer to the soma in sEV-Cap treated-neurons (**Figure 5F**). These results indicated that sEV-Cap and 2D-sEV possessed similar contents and functions but with some differences in the mechanism by which the cytoskeleton was modulated.

These results confirmed that the functionality of sEV-Cap and sEV-2D were equivalent even though the size of the sEV slightly differed between both groups. More important, sEV derived from MenSCs recapitulated the paracrine functions described when the cells themselves are used in the assays.

### sEV-Cap Retain the Immunosuppressive Properties of MenSCs

**Figure 6:**
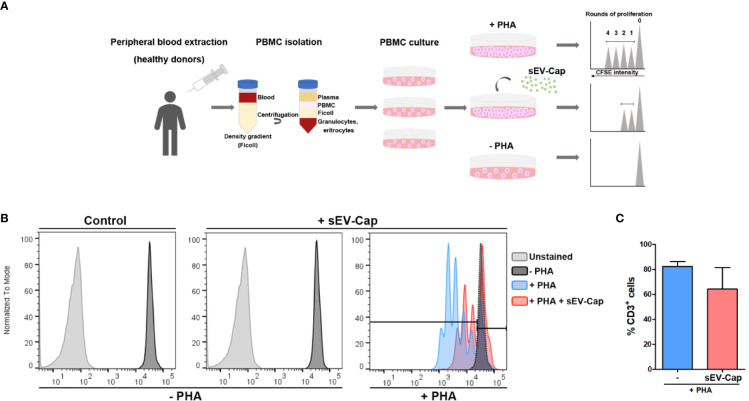
sEV-Cap immunosuppress T lymphocytes proliferation *in vitro*. **(A)** Experimental setup of immunosuppression assay. Blood is obtained from a healthy donor and PBMC (peripheral blood mononuclear cells) are isolated using a density gradient which separated the blood into their components (plasma, mononuclear cells, granulocytes, and red blood cells). The mononuclear fraction contains the population of T lymphocytes (along with B lymphocytes and NK cells). PBMC are dyed with CFSE and maintained in culture under standard conditions. PHA (phycoerythrin) and sEV are added when indicated. After 72 h, PBMC is recovered and stained with CD3 and CD45 antibodies (for the recognition of lymphocytes). **(B)** The dilution of the dye CFSE in mitotic cells is evaluated by flow cytometry. **(C)** 76.4% of PHA-activated T cells proliferate after 72 h but these percentage decreases when sEV-Cap are present up to 52.1% (n = 2).

Finally, we evaluated another classical property of MSCs, which is immunosuppression of T cells in an *in vitro* assay. This role of MSCs represents one of the properties of greatest interest for clinical use in autoimmune diseases. In this assay, PBMC were activated with PHA to induce their proliferation and stained with CFSE. After 72 h, we evaluated the effect of sEV-Cap in the proliferation of T cells measured as a decrease in the number of division cycles (**Figure 6A**). Our data suggest that the presence of sEV inhibited partially T cells PHA-induced proliferation by approximately 30%, supporting the fact that the paracrine properties of MenSCs were maintained in their derived sEV from encapsulated cells (**Figures 6B, C**).

Altogether, these results confirm the rational of using encapsulated cells for the generation and isolation of sEV without time-consuming protocols and with higher purity.

## Discussion

The application of cells or their derivates have been a field of constant growth in modern medicine. Lately, sEV have aroused the interest of researchers due to the innumerable reports showing their biological properties *in vitro* and *in vivo* [reviewed in (Zhang et al., 2019)]. Today there are more than 100 trials registered in www.clinicaltrials.gov in different developmental stages, in which sEV are being tested as treatment in varied pathologies, such as lymphoma, sepsis, wound healing, type I diabetes mellitus, among others, and in another perspective, as diagnostic targets mainly in cancer (such as in lung and pancreatic cancer and squamous cell carcinoma) by analyzing body fluids from patients.

Several techniques have been developed for the isolation of sEV from fluids, tissues, and cell cultures, with different challenges according to the source (Chen B-Y. et al., 2019). However, in this work, we are focused in sEV isolation from cells supernatant.

Multiples reports have shown that the paracrine properties of MSCs can be recapitulated by their secreted vesicles, therefore, one of the current strategies has been to develop new therapies based in MSCs-derived products (Mendt et al., 2019; Yin et al., 2019). The benefits of using encapsulated cells are diverse, from a productive point of view, the use of capsules with a determine pore size determines that the system itself will be cleaner that regular cell media since particles greater than ~200 nm will not be released from the capsules, additionally, capsules can be maintained in bioreactors in order to generate large volumes of supernatant and offers the option of a 3D-culture what can optimize the cells-volume ratio. Additionally, in the case that cell-free agents are needed, encapsulated cells can be used for the production of sEV in order to diminish some steps needed when using ultracentrifugation. However, more research is needed to evaluate differences in the yield of sEV when using encapsulated cells in comparison with cells seeded in a 2D standard fashion.

Additional challenges will be presented for the traceable production of sEV beyond the manufacture process itself. It is well known that there is a high biological variability between different sources of MSCs and also from different donors from the same source (Mendicino et al., 2014; Russell et al., 2015; O’Connor, 2019). This also has been detected in MenSCs and is explained by multiple parameters, such as cell culture conditions, and mostly for the epidemiologic and hormonal background of the donor (Chen L. et al., 2019). Alcayaga et al. reported differences in the CFU-potential and progenitors numbers in MenSCs from 10 different donors (Alcayaga-Miranda et al., 2015a) but differences in this and other parameters are proper of all MSCs and has been shown in multiple publications that even do the variability is a fact, the therapeutic properties of MSCs are also a fact (Galipeau et al., 2016). In the same line, the concept of “potency test” or “potency assay” has become very important in the field of cellular products (Bianco et al., 2013; Deskins et al., 2013; Galipeau et al., 2016; de Wolf et al., 2017), for MenSCs and their derivates. It remains to be determined which test will be the most appropriate considering which properties are of interest for a specific pathology or condition.

Another growing area resides in the intersection between pharmaceutical drugs and cell therapy. This advanced drug delivery resides in loading sEV post-isolation with specifics clinically approved chemical compounds. Recently, evidence has shown that sEV-mediated chemotherapeutic delivery has much improved anti-tumor effects when compared to free drugs in animal tumor models (Wang et al., 2016). As an example, when Paclitaxel was loaded into sEV by sonication, the loaded sEV showed 50 times more anti-tumor effect than free paclitaxel in drug-resistant cancer cells (Kim et al., 2016). The final product will need to meet both cell manufacturing and pharmaceutical industry standards and therefore, requires a homogenous population of particles. Our results show a 7-fold higher presence of sEV (40–200 nm fraction) with a more uniform size distribution, making sEV-Cap a more appropriate protocol for drug-loaded sEV.

From a therapeutic point of view, by using the encapsulated cells system, capsules can be located in the specific tissue where the therapeutic effect is needed, and some complications related with the systemic injection of MSCs can be avoided. Specifically, MSCs which get trapped in a high percentage in the lung microvasculature causing vascular obstructions and the death of the injected cells (Wang et al., 2015). It has been reported that after 48 h of injection, less than 0.1% of total cells may be detected using a high-resolution quantitative 3D imaging system (Schmuck et al., 2016). The high clearance of injected cells and low percentage of MSCs found at the injury site or in non‐target organs can lead to off-target toxicity and overdosing problems. With the localized positioning of capsules, this situation can be prevented, and cloistered cells could maintain a continuous secretion of growth factors and sEV. This point is relevant because sEV are known to have a shorter half-life (Morishita et al., 2017; Göran Ronquist, 2019) compared with their parental cells (Parekkadan and Milwid, 2010; Leibacher and Henschler, 2016) requiring highly repetitive injections to obtain the desired outcome. On the other hand, encapsulated cells secrete sEV continuously but also respond to environmental changes, avoiding undesired effects from multiple injections and from cell byproducts including MVs and apoptotic bodies. Implantation of encapsulated cells producing sEV also allows physical targeting, thereby increasing efficacy as well as acting as a safety device by holding the stem cells at the site needed and physically separating them from the body (Gunzburg and Salmons, 2009). The long-term maintenance of cell viability and the quality of their secretions over time are fundamental questions that remain unanswered. With respect of time, we have previously demonstrated a steady release of 90 µm retrovirus vector particles from encapsulated cells for at least 6 weeks during cell culture and for the same time, during an *in vivo* assay (Saller et al., 2002). Also, a number of different cell lines that have been encapsulated in Cell-in-a-Box have been shown to survive for many weeks to months *in vivo*, and so it might be expected that encapsulated MSCs will present similar survival timeline (Dangerfield et al., 2013).

sEV-Cap were characterized by size, expression of *bona fide* sEV markers, and by their function. With respect to the size, the media size is lower compared to sEV-2D, and this can be explained by the sieve effect of the capsule itself, but we cannot rule out that encapsulated cells secrete smaller vesicles. Lee et al. (2019) have described a subpopulation of sEV inside this fraction, called P100, isolated by an additional ultracentrifugation step after the standard procedure, and with differential functional effects respect to “conventional” sEV or P200 fraction. Interestingly, this P100 fraction express lower levels of CD81, similar to sEV-Cap. This raises the question of whether sEV-Cap are smaller due to the sieve effect of the capsule or are in fact a sub-fraction of the entire sEV production and just the smaller ones are able to be secreted. Further investigation is needed to better define this population and address its biological relevance.

Another theory to explain the smaller size is that the density inside the capsule resembles a confluent cell culture as cells are in close contact in all dimensions. This scenario may affect cellular metabolism and influence the process of formation and secretion of sEV. According to the literature, a high degree confluence induces a major production of sEV (Gurunathan et al., 2019; Thippabhotla et al., 2019) and a decrease in EV secretion (Gudbergsson et al., 2016; Palviainen et al., 2019), and stimulates the secretion of sEV in 3D HeLa cell cultures (Thippabhotla et al., 2019). With respect to the size of sEV, we observed an increase in the abundance of smaller sEV, and some reports indicate that the 3D growth conditions can be the cause (Thippabhotla et al., 2019), and most importantly, sEV shared comparable trophic properties in the assays evaluated here, regardless of their the origin.

Along the same lines, the fate of larger vesicles inside the capsules and their impact on the encapsulated cells needs to be determined. We already mentioned that a high degree of confluency induces a decrease in EV secretion, possibly due to entrapment in the bead (Gudbergsson et al., 2016; Palviainen et al., 2019). In accordance with the mentioned data, we suspect that encapsulated cells sense an increase in the concentration of EV inside the capsule which can activate some auto-regulatory pathways that inhibits the generation of EV. As sEV freely diffuse from the capsules, this process might not be inhibited, but more research is needed to understand this phenomenon.

Nevertheless, in summary, we have successfully showed a novel, less expensive, and faster method to generate sEV from MenSCs. Due to its simplicity, it is possible to assemble the protocol under GMP conditions, since (i) we already confirmed the feasibility of isolating MenSCs for clinical use and (ii) GMP production for Cell-in-a-Box has already been established. Moreover, encapsulated cells may be used as a device for releasing sEV *in vivo* constantly, until the capsules are removed. Finally, particles produced under the encapsulation protocol display advantageous properties positioning them as prominent vehicles for drug-loaded exosome strategies.

## Data Availability Statement

All datasets generated for this study are included in the article/**Supplementary Material**.

## Ethics Statement

The studies involving human participants were reviewed and approved by The Ethics Committee of Universidad de los Andes. The patients/participants provided their written informed consent to participate in this study.

## Author Contributions

M-PR and MK were responsible for conception, design and data analysis. M-PR, GZ, PC, MH, and AF-V were responsible for data acquisition and analysis. GZ and MK were responsible for interpretation of the results and manuscript writing. PT performed the cell encapsulations and BS, JD, and WG were responsible for cell encapsulation experiment planning and data analysis. PC and UW performed the neurite growth assays. All authors read and approved the final manuscript.

## Funding

This study was cofunded by the Consorcio REGENERO, Chilean Biotechnology Company and Austrianova.

## Conflict of Interest

GZ, AF-V, and M-PR receive stipends from Consorcio REGENERO, a Chilean Biotechnology Company, for cell therapy development. MK receives stipends from Cells for Cells, a Chilean Biotechnology Company, for cell therapy development. JD, BS, WG, and PT receive stipends from Austrianova Singapore Pte Ltd, biotech company which develops several cell-based products and among them is the cell encapsulation technology, patented and marketed as a kit.

The remaining authors declare that the research was conducted in the absence of any commercial or financial relationships that could be construed as a potential conflict of interest.

## Supplementary Material

Supplementary Figure 1 NTA report for EV.

Supplementary Figure 2 NTA report for sEV-Cap.

Supplementary Figure 3 NTA report for sEV-2D.

Supplementary Figure 4 Flow cytometry histograms derived from the data showed as dot blots.

Supplementary Figure 5 Uptake analysis. **(A)** PKH26 stained sEV-Cap (red) are effectively taken up by MenSCs monolayers. **(B)** Quantification of PKH26+ cells compared to PBS control, *P < 0.05 unpaired Student’s t test.

## Abbreviations

MenSCs, menstrual blood-derived stem cells; sEV, small extracellular vesicles; NTA, nanoparticle tracking analysis; EV, extracellular vesicles; MSCs, mesenchymal stem cells; MV, microvesicles; TEM, transmission electron microscopy; PBMC, peripheral blood mononuclear cells; PHA, phytohemagglutinin.

## References

[B1] Acarregui A. Orive G. Pedraz J. L. Hernández R. M. (2013). Therapeutic Applications of Encapsulated Cells (Totowa, NJ: Humana Press), 349–364. 10.1007/978-1-62703-550-7_23 23934816

[B2] Alcayaga-Miranda F. Cuenca J. Luz-Crawford P. Aguila-Díaz C. Fernandez A. Figueroa F. E. (2015a). Characterization of menstrual stem cells: angiogenic effect, migration and hematopoietic stem cell support in comparison with bone marrow mesenchymal stem cells. Stem Cell Res. Ther. 6, 32. 10.1186/s13287-015-0013-5 25889741 PMC4404686

[B3] Alcayaga-Miranda F. Cuenca J. Martin A. Contreras L. Figueroa F. E. Khoury M. (2015b). Combination therapy of menstrual derived mesenchymal stem cells and antibiotics ameliorates survival in sepsis. Stem Cell Res. Ther. 6, 199. 10.1186/s13287-015-0192-0 26474552 PMC4609164

[B4] Bianco P. Cao X. Frenette P. S. Mao J. J. Robey P. G. Simmons P. J. (2013). The meaning, the sense and the significance: translating the science of mesenchymal stem cells into medicine. Nat. Med. 19, 35–42. 10.1038/nm.3028 23296015 PMC3998103

[B5] Chen B.-Y. Sung C. W.-H. Chen C. Cheng C.-M. Lin D. P.-C. Huang C.-T. (2019). Advances in exosomes technology. Clin. Chim Acta 493, 14–19. 10.1016/j.cca.2019.02.021 30797770

[B6] Chen L. Qu J. Xiang C. (2019). The multi-functional roles of menstrual blood-derived stem cells in regenerative medicine. Stem Cell Res. Ther. 10, 1. 10.1186/s13287-018-1105-9 30606242 PMC6318883

[B7] Dangerfield J. A. Salmons B. Corteling R. Abastado J.-P. Sinden J. H. Gunzburg W. (2013). “The Diversity of Uses for Cellulose Sulphate Encapsulation,” in Bioencapsulation Living Cells Divers Medical Applications. Eds. A. Dangerfield J. Salmons B. Corteling R. Abastado J.-P. Sinden J. H. Gunzburg W. (Bentham Science Publishers;), 70–92. 10.2174/9781608057207113010006

[B8] Dautzenberg H. Schuldt U. Grasnick G. Karle P. Müller P. Löhr M. (1999). Development of Cellulose Sulfate-based Polyelectrolyte Complex Microcapsules for Medical Applications. Ann. N Y Acad. Sci. 875, 46–63. 10.1111/j.1749-6632.1999.tb08493.x 10415557

[B9] de Wolf C. van de Bovenkamp M. Hoefnagel M. (2017). Regulatory perspective on in vitro potency assays for human mesenchymal stromal cells used in immunotherapy. Cytotherapy. 19, 784–797. 10.1016/j.jcyt.2017.03.076 28457740

[B10] Deskins D. L. Bastakoty D. Saraswati S. Shinar A. Holt G. E. Young P. P. (2013). Human Mesenchymal Stromal Cells: Identifying Assays to Predict Potency for Therapeutic Selection. Stem Cells Transl. Med. 2, 151–158. 10.5966/sctm.2012-0099 23362238 PMC3659751

[B11] Emerich D. F. Orive G. Thanos C. Tornoe J. Wahlberg L. U. (2014). Encapsulated cell therapy for neurodegenerative diseases: From promise to product. Adv. Drug Delivery Rev. 67–68, 131–141. 10.1016/j.addr.2013.07.008 23880505

[B12] Göran Ronquist K. (2019). Extracellular vesicles and energy metabolism. Clin. Chim Acta 488, 116–121. 10.1016/j.cca.2018.10.044 30395864

[B13] Galipeau J. Krampera M. Barrett J. Dazzi F. Deans R. J. DeBruijn J. (2016). International Society for Cellular Therapy perspective on immune functional assays for mesenchymal stromal cells as potency release criterion for advanced phase clinical trials. Cytotherapy. 18, 151–159. 10.1016/j.jcyt.2015.11.008 26724220 PMC4745114

[B14] Gardiner C. Di Vizio D. Sahoo S. Théry C. Witwer K. W. Wauben M. (2016). Techniques used for the isolation and characterization of extracellular vesicles: results of a worldwide survey. J. Extracell Vesicles. 5, 32945. 10.3402/jev.v5.32945 27802845 PMC5090131

[B15] González P. L. Carvajal C. Cuenca J. Alcayaga-Miranda F. Figueroa F. E. Bartolucci J. (2015). Chorion Mesenchymal Stem Cells Show Superior Differentiation, Immunosuppressive, and Angiogenic Potentials in Comparison With Haploidentical Maternal Placental Cells. Stem Cells Transl. Med. 4, 1109–1121. 10.5966/sctm.2015-0022 26273064 PMC4572900

[B16] Gonzalez-Pujana A. Santos E. Orive G. Pedraz J. L. Hernandez R. M. (2017). Cell microencapsulation technology: Current vision of its therapeutic potential through the administration routes. J. Drug Delivery Sci. Technol. 42, 49–62. 10.1016/j.jddst.2017.03.028

[B17] Gudbergsson J. M. Johnsen K. B. Skov M. N. Duroux M. (2016). Systematic review of factors influencing extracellular vesicle yield from cell cultures. Cytotechnology. 68, 579–592. 10.1007/s10616-015-9913-6 26433593 PMC4960200

[B18] Gunzburg W. H. Salmons B. (2009). Stem cell therapies: on track but suffer setback. Curr. Opin. Mol. Ther. 11, 360–363. 19649980

[B19] Gurunathan S. Kang M.-H. Jeyaraj M. Qasim M. Kim J.-H. (2019). Review of the Isolation, Characterization, Biological Function, and Multifarious Therapeutic Approaches of Exosomes. Cells. 8, 307. 10.3390/cells8040307 PMC6523673 30987213

[B20] Han C. Zhou J. Liang C. Liu B. Pan X. Zhang Y. (2019). Human umbilical cord mesenchymal stem cell derived exosomes encapsulated in functional peptide hydrogels promote cardiac repair. Biomater Sci. 7, 2920–2933. 10.1039/C9BM00101H 31090763

[B21] Kaech S. Banker G. (2006). Culturing hippocampal neurons. Nat. Protoc. 1, 2406–2415. 10.1038/nprot.2006.356 17406484

[B22] Killer M. C. Nold P. Henkenius K. Fritz L. Riedlinger T. Barckhausen C. (2017). Immunosuppressive capacity of mesenchymal stem cells correlates with metabolic activity and can be enhanced by valproic acid. Stem Cell Res. Ther. 8, 100. 10.1186/s13287-017-0553-y 28446224 PMC5406996

[B23] Kim M. S. Haney M. J. Zhao Y. Mahajan V. Deygen I. Klyachko N. L. (2016). Development of exosome-encapsulated paclitaxel to overcome MDR in cancer cells. Nanomed. Nanotechnol. Biol. Med. 12, 655–664. 10.1016/j.nano.2015.10.012 PMC4809755 26586551

[B24] Löhr J. M. Haas S. L. Kröger J. C. Friess H. M. Höft R. Goretzki P. E. (2014). Encapsulated cells expressing a chemotherapeutic activating enzyme allow the targeting of subtoxic chemotherapy and are safe and efficacious: data from two clinical trials in pancreatic cancer. Pharmaceutics. 6, 447–466. 10.3390/pharmaceutics6030447 25116885 PMC4190529

[B25] Lee S.-S. Won J.-H. Lim G. J. Han J. Lee J. Y. Cho K.-O. (2019). A novel population of extracellular vesicles smaller than exosomes promotes cell proliferation. Cell Commun. Signal. 17, 95. 10.1186/s12964-019-0401-z 31416445 PMC6694590

[B26] Leibacher J. Henschler R. (2016). Biodistribution, migration and homing of systemically applied mesenchymal stem/stromal cells. Stem Cell Res. Ther. 7, 7. 10.1186/s13287-015-0271-2 26753925 PMC4709937

[B27] Li P. Kaslan M. Lee S. H. Yao J. Gao Z. (2017). Progress in Exosome Isolation Techniques. Theranostics. 7, 789–804. 10.7150/thno.18133 28255367 PMC5327650

[B28] Liu X. Yang Y. Li Y. Niu X. Zhao B. Wang Y. (2017). Integration of stem cell-derived exosomes with in situ hydrogel glue as a promising tissue patch for articular cartilage regeneration. Nanoscale 9, 4430–4438. 10.1039/C7NR00352H 28300264

[B29] Margolis L. Sadovsky Y. (2019). The biology of extracellular vesicles: The known unknowns. PloS Biol. 17, e3000363. 10.1371/journal.pbio.3000363 31318874 PMC6667152

[B30] Mendicino M. Bailey A. M. Wonnacott K. Puri R. K. Bauer S. R. (2014). MSC-based product characterization for clinical trials: an FDA perspective. Cell Stem Cell. 14, 141–145. 10.1016/j.stem.2014.01.013 24506881

[B31] Mendt M. Rezvani K. Shpall E. (2019). Mesenchymal stem cell-derived exosomes for clinical use. Bone Marrow Transplant. 54, 789–792. 10.1038/s41409-019-0616-z 31431712

[B32] Meng X. Ichim T. E. Zhong J. Rogers A. Yin Z. Jackson J. (2007). Endometrial regenerative cells: a novel stem cell population. J. Transl. Med. 5, 57. 10.1186/1479-5876-5-57 18005405 PMC2212625

[B33] Michałowska M. Winiarczyk S. Adaszek Ł Łopuszyński W. Grądzki Z. Salmons B. (2014). Phase I/II clinical trial of encapsulated, cytochrome P450 expressing cells as local activators of cyclophosphamide to treat spontaneous canine tumours. PloS One 9, e102061. 10.1371/journal.pone.0102061 25028963 PMC4100764

[B34] Morishita M. Takahashi Y. Nishikawa M. Takakura Y. (2017). Pharmacokinetics of Exosomes—An Important Factor for Elucidating the Biological Roles of Exosomes and for the Development of Exosome-Based Therapeutics. J. Pharm. Sci. 106, 2265–2269. 10.1016/j.xphs.2017.02.030 28283433

[B35] O’Connor K. C. (2019). Molecular Profiles of Cell-to-Cell Variation in the Regenerative Potential of Mesenchymal Stromal Cells. Stem Cells Int. 2019, 1–14. 10.1155/2019/5924878 PMC6766122 31636675

[B36] Orlando G. Gianello P. Salvatori M. Stratta R. J. Soker S. Ricordi C. (2014). Cell Replacement Strategies Aimed at Reconstitution of the β-Cell Compartment in Type 1 Diabetes. Diabetes 63, 1433–1444. 10.2337/db13-1742 24757193

[B37] Ortner V. Kaspar C. Halter C. Töllner L. Mykhaylyk O. Walzer J. (2012). Magnetic field-controlled gene expression in encapsulated cells. J. Control Release. 158, 424–432. 10.1016/j.jconrel.2011.12.006 22197778 PMC3329627

[B38] Palviainen M. Saari H. Kärkkäinen O. Pekkinen J. Auriola S. Yliperttula M. (2019). Metabolic signature of extracellular vesicles depends on the cell culture conditions. J. Extracell Vesicles. 8, 1596669. 10.1080/20013078.2019.1596669 31007875 PMC6461113

[B39] Parekkadan B. Milwid J. M. (2010). Mesenchymal stem cells as therapeutics. Annu. Rev. BioMed. Eng. 12, 87–117. 10.1146/annurev-bioeng-070909-105309 20415588 PMC3759519

[B40] Pegtel D. M. Gould S. J. (2019). Exosomes. Annu. Rev. Biochem. Annu. Rev. 88, 487–514. 10.1146/annurev-biochem-013118-111902 31220978

[B41] Rosenberger L. Ezquer M. Lillo-Vera F. Pedraza P. L. Ortúzar M. I. González P. L. (2019). Stem cell exosomes inhibit angiogenesis and tumor growth of oral squamous cell carcinoma. Sci. Rep. 9, 663. 10.1038/s41598-018-36855-6 30679544 PMC6345809

[B42] Russell A. L. Glover L. E. Zubair A. (2015). Impact of tissue origin, donor and culture conditions on mesenchymal stem cell characteristics. Cytotherapy. 17, S81. 10.1016/j.jcyt.2015.03.589

[B43] Saller R. M. Indraccolo S. Coppola V. Esposito G. Stange J. Mitzner S. (2002). Encapsulated cells producing retroviral vectors for *in vivo* gene transfer. J. Gene Med. 4, 150–160. 10.1002/jgm.257 11933216

[B44] Salmons B. Gunzburg W. H. (2018). Release characteristics of cellulose sulphate capsules and production of cytokines from encapsulated cells. Int. J. Pharm. 548, 15–22. 10.1016/j.ijpharm.2018.06.040 29933063

[B45] Samsonraj R. M. Raghunath M. Nurcombe V. Hui J. H. van Wijnen A. J. Cool S. M. (2017). Concise Review: Multifaceted Characterization of Human Mesenchymal Stem Cells for Use in Regenerative Medicine. Stem Cells Transl. Med. 6, 2173–2185. 10.1002/sctm.17-0129 29076267 PMC5702523

[B46] Schmuck E. G. Koch J. M. Centanni J. M. Hacker T. A. Braun R. K. Eldridge M. (2016). Biodistribution and Clearance of Human Mesenchymal Stem Cells by Quantitative Three-Dimensional Cryo-Imaging After Intravenous Infusion in a Rat Lung Injury Model. Stem Cells Transl. Med. 5, 1668–1675. 10.5966/sctm.2015-0379 27460855 PMC5189648

[B47] Shi Q. Qian Z. Liu D. Sun J. Wang X. Liu H. (2017). GMSC-Derived Exosomes Combined with a Chitosan/Silk Hydrogel Sponge Accelerates Wound Healing in a Diabetic Rat Skin Defect Model. Front. Physiol. 8, 904. 10.3389/fphys.2017.00904 29163228 PMC5681946

[B48] Théry C. Witwer K. W. Aikawa E. Alcaraz M. J. Anderson J. D. Andriantsitohaina R. (2018). Minimal information for studies of extracellular vesicles 2018 (MISEV2018): a position statement of the International Society for Extracellular Vesicles and update of the MISEV2014 guidelines. J. Extracell Vesicles. 7, 1535750. 10.1080/20013078.2018.1535750 30637094 PMC6322352

[B49] Thippabhotla S. Zhong C. He M. (2019). 3D cell culture stimulates the secretion of in vivo like extracellular vesicles. Sci. Rep. 9, 13012. 10.1038/s41598-019-49671-3 31506601 PMC6736862

[B50] Wang S. Guo L. Ge J. Yu L. Cai T. Tian R. (2015). Excess Integrins Cause Lung Entrapment of Mesenchymal Stem Cells. Stem Cells 33, 3315–3326. 10.1002/stem.2087 26148841

[B51] Wang J. Zheng Y. Zhao M. (2016). Exosome-Based Cancer Therapy: Implication for Targeting Cancer Stem Cells. Front. Pharmacol. 7, 533. 10.3389/fphar.2016.00533 28127287 PMC5226951

[B52] Wang C. Wang M. Xu T. Zhang X. Lin C. Gao W. (2019). Engineering Bioactive Self-Healing Antibacterial Exosomes Hydrogel for Promoting Chronic Diabetic Wound Healing and Complete Skin Regeneration. Theranostics. 9, 65–76. 10.7150/thno.29766 30662554 PMC6332800

[B53] Yin K. Wang S. Zhao R. C. (2019). Exosomes from mesenchymal stem/stromal cells: a new therapeutic paradigm. Biomark Res. 7, 8. 10.1186/s40364-019-0159-x 30992990 PMC6450000

[B54] Zhang Y. Liu Y. Liu H. Tang W. H. (2019). Exosomes: biogenesis, biologic function and clinical potential. Cell Biosci. 9, 19. 10.1186/s13578-019-0282-2 30815248 PMC6377728

