# Supplementary figures and images for: Semipermeable Cellulose Beads Allow Selective and Continuous Release of Small Extracellular Vesicles (sEV) From Encapsulated Cells
Source: Front Pharmacol. 2020 May 21;11:679. doi: 10.3389/fphar.2020.00679 (PMC7253686; doi:10.3389/fphar.2020.00679)

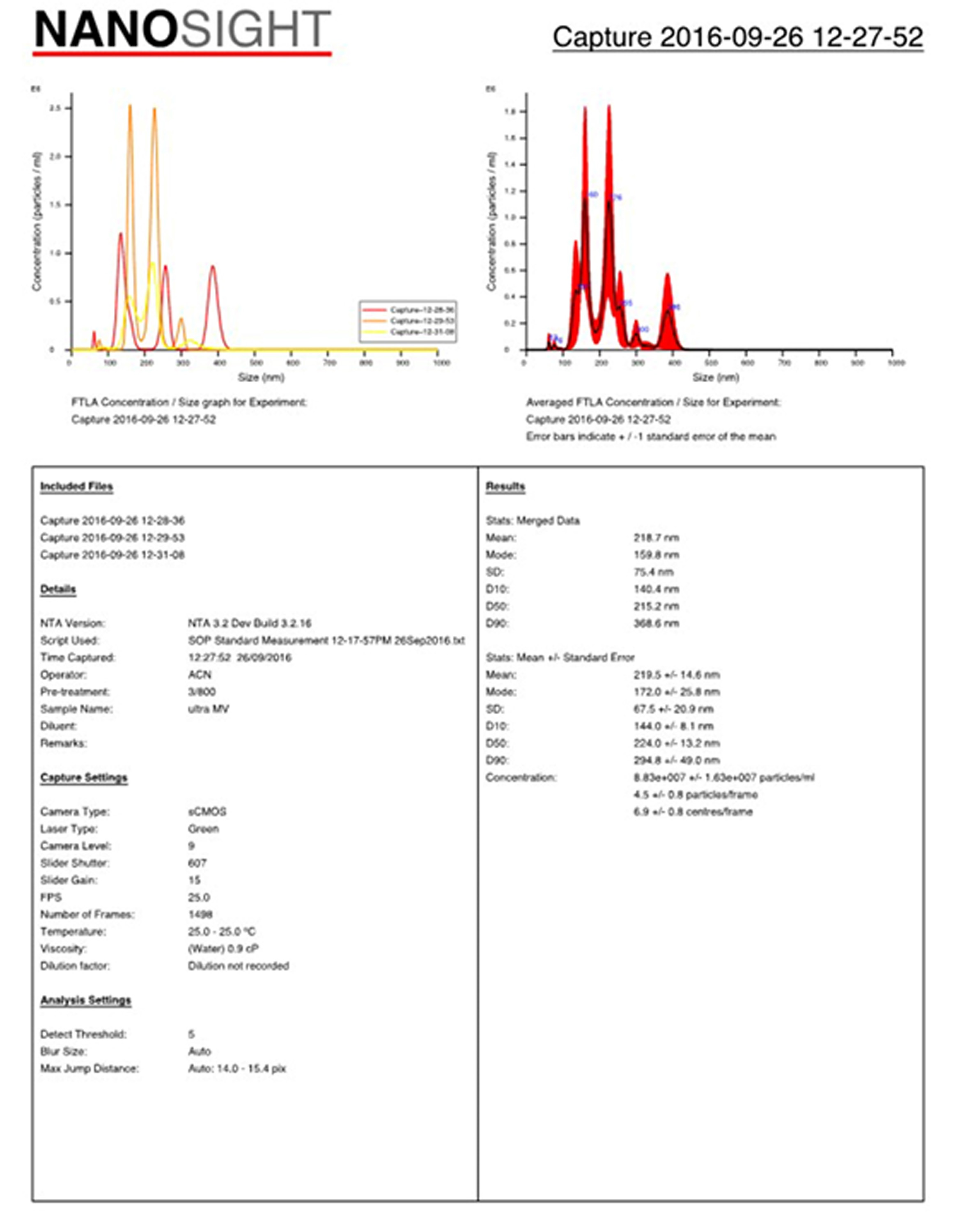

Supplement: Supplementary Figure 1 — NTA report for EV. [file Image_1.jpeg]

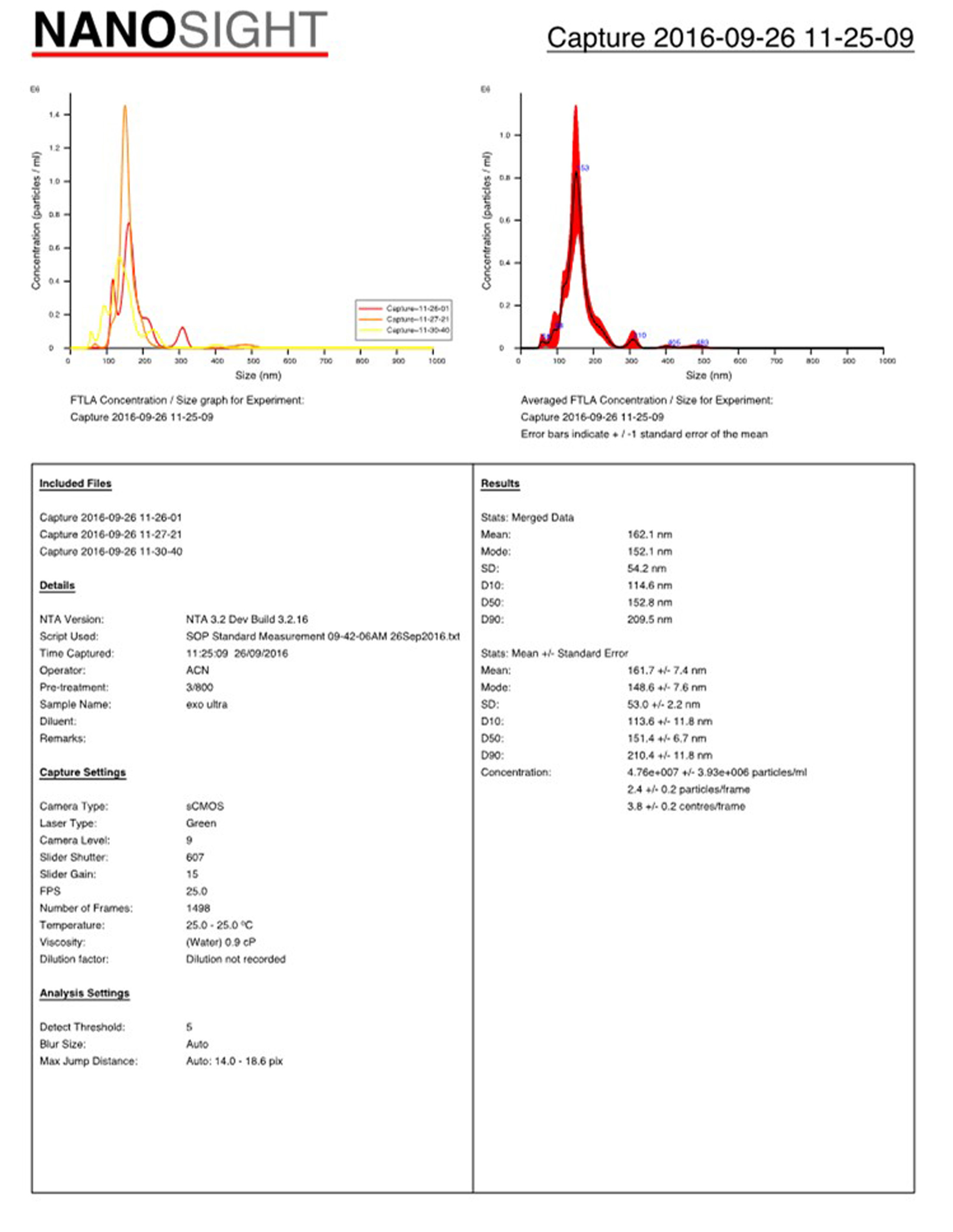

Supplement: Supplementary Figure 2 — NTA report for sEV-Cap. [file Image_2.jpeg]

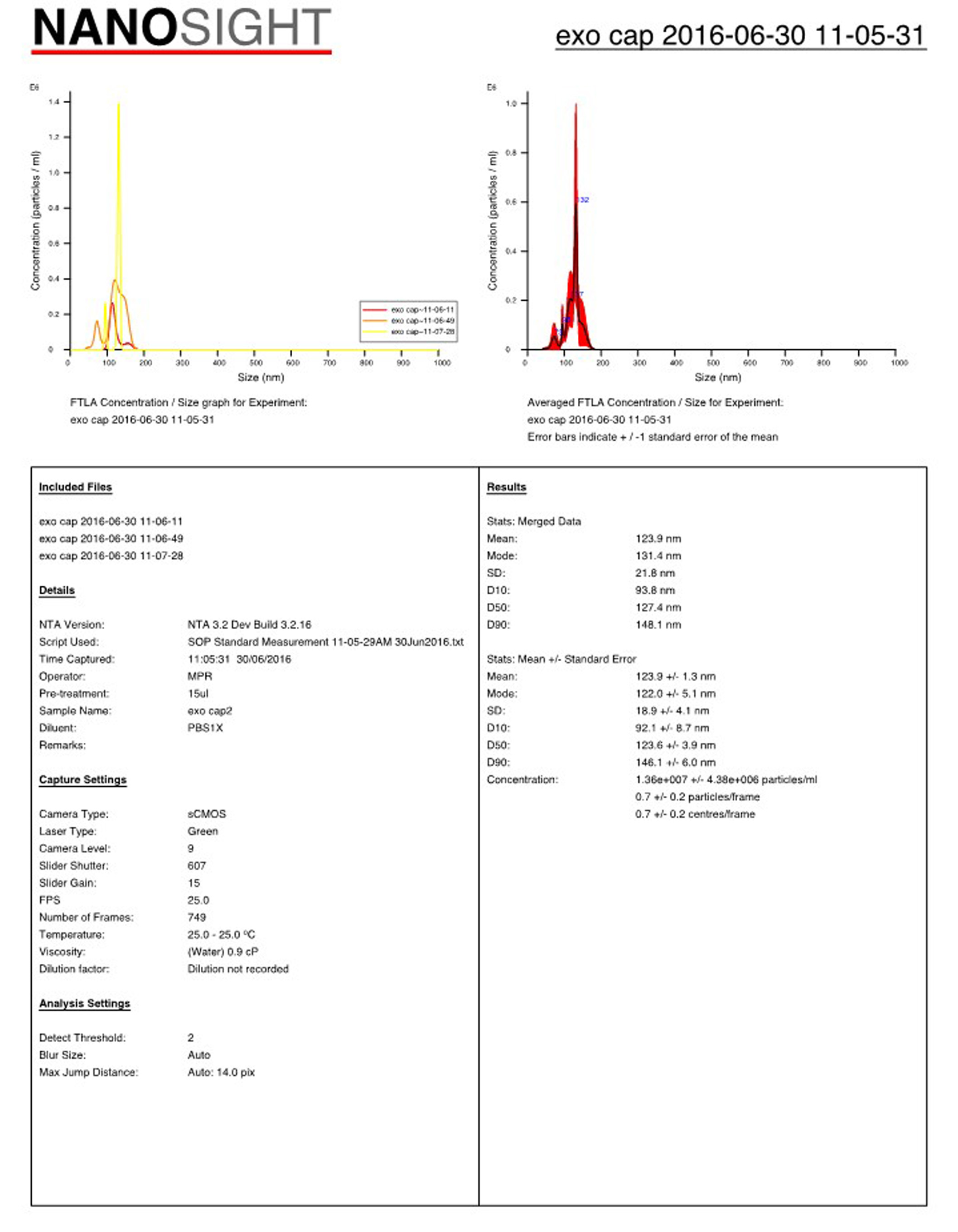

Supplement: Supplementary Figure 3 — NTA report for sEV-2D. [file Image_3.jpeg]

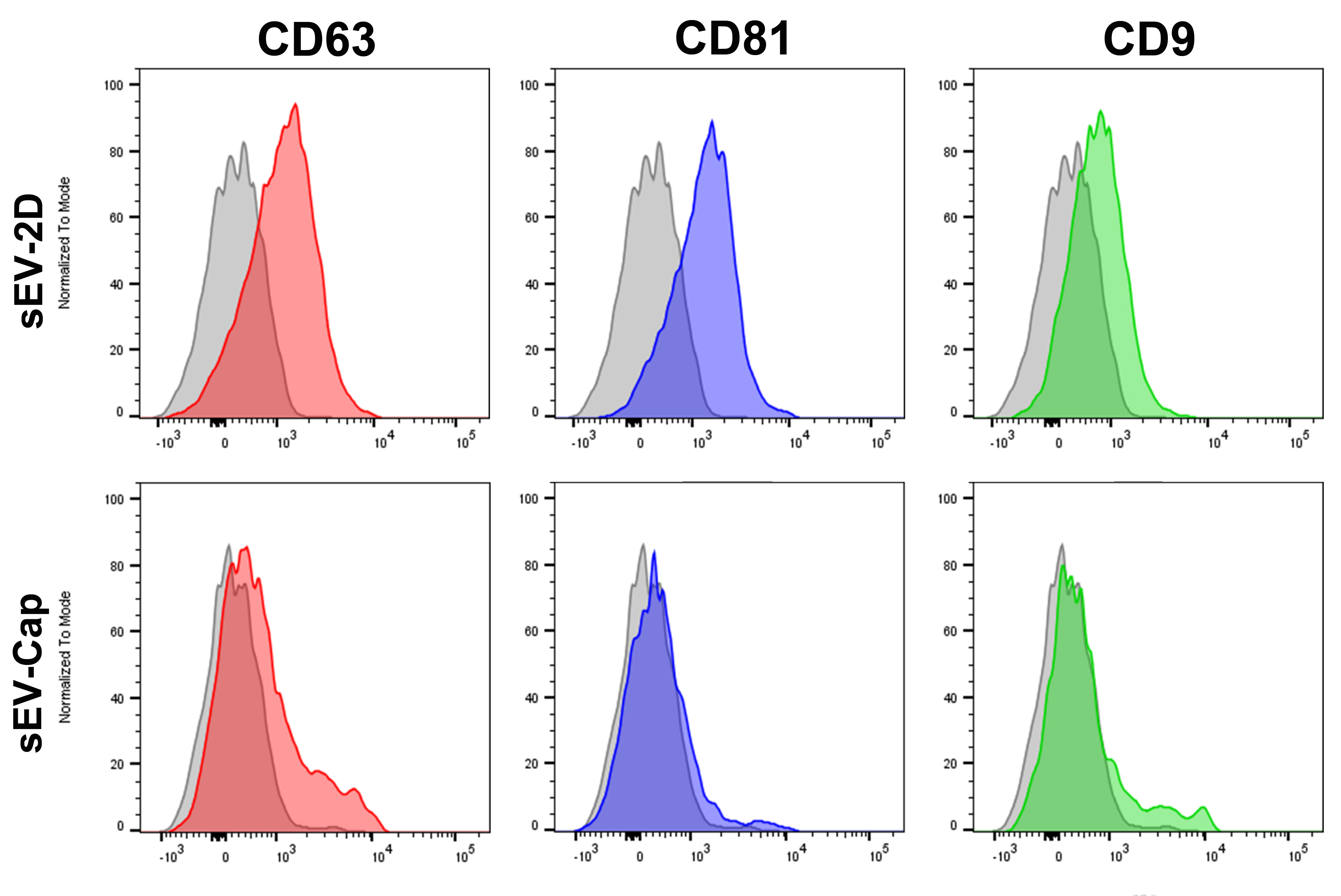

Supplement: Supplementary Figure 4 — Flow cytometry histograms derived from the data showed as dot blots. [file Image_4.jpeg]

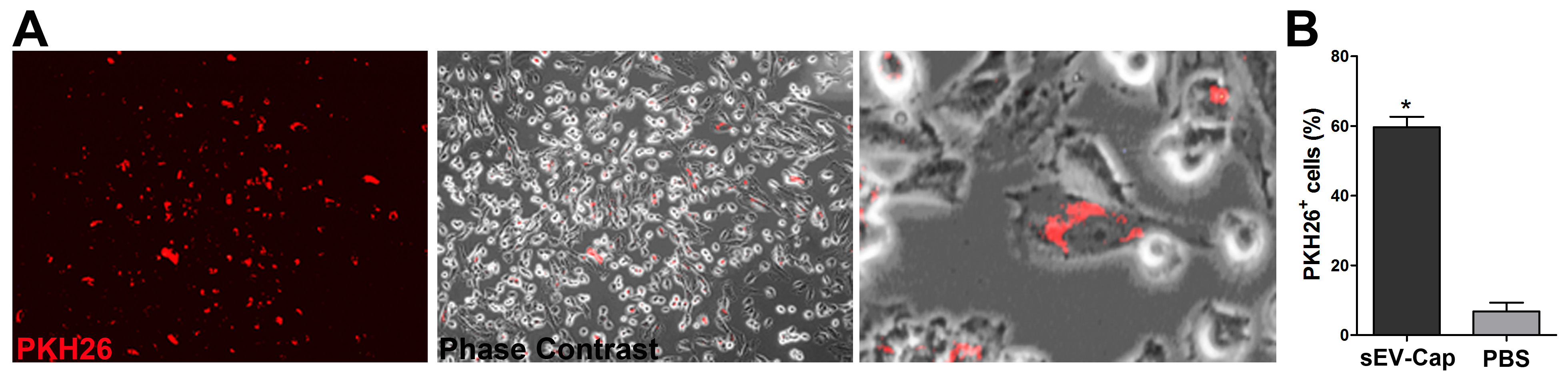

Supplement: Supplementary Figure 5 — Uptake analysis. (A) PKH26 stained sEV-Cap (red) are effectively taken up by MenSCs monolayers. (B) Quantification of PKH26+ cells compared to PBS control, *P < 0.05 unpaired Student’s t test. [file Image_5.jpeg]
